# Hypothalamic Leptin Resistance: From BBB to BBSome

**DOI:** 10.1371/journal.pgen.1005980

**Published:** 2016-05-05

**Authors:** Miguel López

**Affiliations:** 1 NeurObesity Group, Department of Physiology, CIMUS, University of Santiago de Compostela-Instituto de Investigación Sanitaria, Santiago de Compostela, Spain; 2 CIBER Fisiopatología de la Obesidad y Nutrición (CIBERobn), Santiago de Compostela, Spain; The Hospital for Sick Children, University of Toronto, CANADA

In 1969, Douglas Coleman joined the bodies of an obese and a normal mouse by a procedure called parabiosis. Days later, the normal mouse starved. Something had taken away its desire to eat [1]. It was not until almost 25 years later that the true nature of what Coleman then called "satiety factor" was revealed. In 1994, Jeffrey Friedman identified the adipocytic hormone leptin [2,3] and it was made official: obesity has a genetic background.

Leptin binds and activates a receptor of the cytokine receptor family. Alternative mRNA splicing and posttranslational processing result in several receptor isoforms (LRa, LRb, LRc, LRe, and LRf); the long isoform, LRb, is implicated in signal transduction. The other isoforms may act as leptin sequesters and transporters, binding leptin without signal transduction. LRb possesses a long intracellular domain that binds to Janus kinase 2 (JAK2) and to signal transducers and activators of transcription (STAT)-3 and STAT-5. Plasma leptin crosses the blood–brain barrier (BBB) via a saturable process, reaching the hypothalamus, where LRb is widely expressed. Acting in the hypothalamic arcuate nucleus (ARC), leptin inhibits the expression of orexigenic neuropeptides (e.g., agouti-related protein [AgRP], and neuropeptide Y [NPY]) and increases the expression of anorexigenic neuropeptides (e.g., proopiomelanocortin [POMC]), which decreases feeding and increases energy expenditure [4,5].

The role of leptin in human obesity remains poorly understood. Leptin deficiency and mutations in leptin receptor cause morbid obesity; however, these defects are extremely rare [6]. On the contrary, the majority of obese humans have high levels of leptin, suggesting leptin insensitivity or resistance [4]. Central leptin resistance may develop via different mechanisms (Fig 1), such as (1) impairment in the function of the saturable leptin transporters in the BBB [7]; (2) deficient leptin signaling [4,5,7,8]; (3) lipotoxicity and endoplasmic reticulum (ER) stress, as well as (4) inflammatory signals that have also been shown to modulate leptin responsiveness [9]. Despite this evidence, recent data questioned that obesity may be a result of leptin resistance [10].

**Fig 1:**
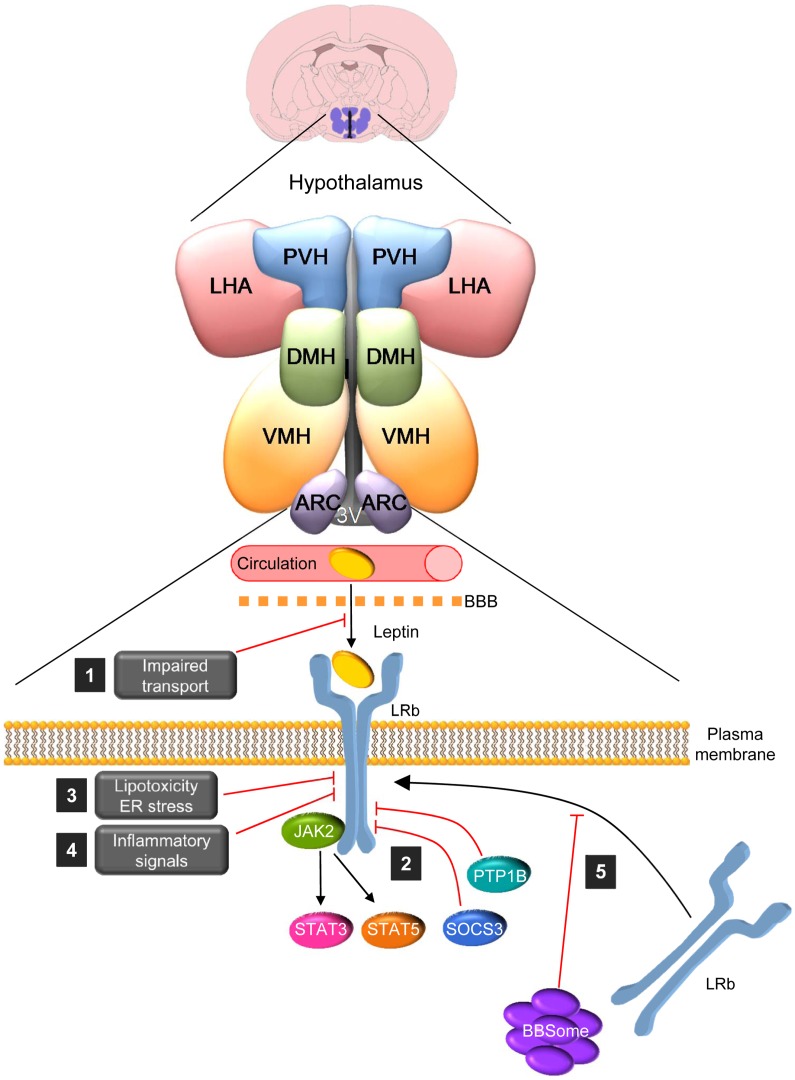
The BBsome regulates hypothalamic leptin resistance. Leptin binding to leptin receptor (LRb) dimmers promotes the phosphorylation of JAK2, which phosphorylates tyrosine residues on the intracellular part of LRb. Each of these phosphorylated residues recruits an exclusive set of downstream signaling molecules, such as signal transducers and activators of transcription 3 and 5 (STAT-3 and STAT-5). Leptin resistance may develop via different mechanisms, such as (1) impairment of the leptin transport in the BBB; (2) deficient leptin signaling due, for example, to decreased LRb expression, suppressor of cytokine signaling-3 (SOCS-3), or protein tyrosine phosphatase 1B (PTP1B); (3) lipotoxicity and ER stress; (4) inflammatory signals; and (5) in a recent issue of *PLOS Genetics*, Rahmouni and colleagues describe a novel mechanism that promotes leptin resistance: impaired LRb trafficking to the plasma membrane in cells due to defects in the BBSome, a protein complex encoded by genes that, when mutated, cause the Bardet-Biedl Syndrome (BBS). 3V: third ventricle; ARC: arcuate nucleus of the hypothalamus; DMH; dorsomedial nucleus of the hypothalamus; LHA: lateral hypothalamic area; PVH: paraventricular nucleus of the hypothalamus; VMH: ventromedial nucleus of the hypothalamus.

In a recent publication in *PLOS Genetics*, Kamal Rahmouni and colleagues describe a novel mechanism that promotes leptin resistance. They show that neuronal *Bardet-Biedl Syndrome* (BBS) proteins influence energy homeostasis through the control of cell surface expression of the leptin receptor [11]. BBS is a rare and highly pleiotropic autosomal recessive disorder characterized by retinal dystrophy, polydactyly, renal and gonadal anomalies, cognitive impairment, and obesity [12,13]. BBS is part of an emerging class of diseases named ciliopathies that are characterized by disorders of the cellular cilia, anchoring structures, basal bodies, or impaired ciliary function [12]. At least 20 genes (BBS1-BBS20), when mutated, lead to defective cilia and result in BBS [12,13]. Eight of the BBS proteins (BBS1, BBS2, BBS4, BBS5, BBS7, BBS8, BBS9, and BBS18) form the stable BBSome complex, which mediates protein trafficking to the ciliary membrane and, perhaps, to other membrane compartments [12,13].

BBS mouse models recapitulate many of the characteristics found in patients, including obesity, which is associated with hyperphagia and leptin resistance. Notably, some mice showed altered hypothalamic expression of POMC [14], indicating that the primary effect leading to positive energy balance might imply altered central leptin action. To address that hypothesis, Rahmouni and colleagues generated mice with genetic disruption of BBS proteins selectively in the central nervous system (CNS), in the hypothalamus, and in LRb-expressing cells. The crossing of *Bbs1* floxed with *Nestin Cre* mice led to animals with BBS1 deficiency in the brain, but not in peripheral tissues. These mice showed an age- and gender-dependent increase in body weight and adiposity, hyperleptinemia, and hyperphagia, as well as reduced hypothalamic expression of POMC and decreased energy expenditure. Next, they generated mice models lacking BB1 protein in LRb-expressing cells by crossing *Bbs1* floxed with *LRb Cre* mice. Moreover, they ablated *Bbs1* in the mediobasal hypothalamus (MBH) by postnatal virogenetic targeting of *Bbs1* floxed mice with adeno-associated viruses encoding Cre. Remarkably, *Bbs1* gene ablation from the LRb-expressing cells or in the MBH was sufficient to cause to obesity in mice. The coexistence of obesity and hyperleptinemia in *Nestin*^*cre*^*/Bbs*^*fl/fl*^ and *LRb*^*cre*^*/Bbs*^*fl/fl*^ mice was suggestive of leptin resistance, which was functionally confirmed. The authors also examined the contribution of obesity to leptin resistance in *LRb*^*cre*^*/Bbs*^*fl/fl*^ mice by administering leptin to calorie-restricted or young (non-obese) animals. Their data showed that both models exhibit a substantially reduced response to leptin, indicating that leptin resistance in mice with ablated *Bbs1* gene in LRb-expressing cells is not a result of obesity. Further analysis showed that neither decreased LRb expression nor impaired signaling was the mechanism leading to leptin resistance.

One important feature of *LRb*^*cre*^*/Bbs*^*fl/fl*^ mice was that deletion of the *Bbs1* gene did not impact cilia in LRb cells, suggesting that it did not account for the leptin resistance. To test that possibility, the authors ablated the intraflagellar transport 88 (*Ift88*) gene, a key factor for ciliogenesis [12,13]. The *LRb*^*cre*^*/Ift88* ^*fl/fl*^ mice displayed a slight feeding-independent weight gain, but had a normal response to leptin. This important finding indicated that LRb-independent mechanisms are involved in the obesity associated with ciliopathies other than BBS. An interesting possibility for leptin resistance could be through BBS protein trafficking [12,13]. By using in vivo and in vitro models, they demonstrated that knockdown of BBS1 or BBS2, but not IFT88, reduced the levels of LRb (but not LRa) in the plasma membrane, leading to decreased leptin signaling. Overall, these data indicate that selective disruption of BBS proteins impairs the transport of leptin receptor to the plasma membrane, promoting leptin resistance and obesity (Fig 1) [11].

The significance of these data relates to several novel findings. Firstly, Rahmouni and colleagues described a new pathological mechanism leading to obesity, specifically impaired LRb trafficking in hypothalamic cells and leptin resistance due to defects in the BBSome, independently of cilia and obesity. In this sense, although, some data had implicated other cilia-related proteins in the regulation of energy balance and the participation of leptin signaling was not fully demonstrated [15]. Secondly, these data change the paradigm that BBS proteins are only associated with ciliary function because they regulate receptor trafficking through the BBSome as a mechanism modulating hormonal actions. This idea is also reinforced by recent data demonstrating that disruption of BBS proteins interferes with insulin receptor at the cell surface, leading to altered glucose metabolism [16]. Thirdly, and more importantly, this evidence provides the molecular basis for the obesity associated with BBS patients. This is highly relevant. In fact, there are just a few known examples of primary hypothalamic obesity in humans, such as hypothalamic injuries [17], and defects in the melanocortin system, such as POMC and *mc4r* (melanocortin receptor 4) gene mutants [6].

In summary, the new study of Rahmouni and colleagues establishes the role of disrupted BBS proteins as a mechanism underlying leptin resistance due to impaired transport of the leptin receptor to the plasma membrane; more importantly, they explain obesity in BBS patients [11]. Overall, this important information has clear translational repercussions, as it may provide new strategies to ameliorate some of the symptoms of this devastating disease.

## References

[1] Coleman DL, Hummel KP (1969) Effects of parabiosis of normal with genetically diabetic mice. Am J Physiol 217: 1298–1304. 5346292 10.1152/ajplegacy.1969.217.5.1298

[2] Zhang Y, Proenca R, Maffei M, Barone M, Leopold L et al (1994) Positional cloning of the mouse obese gene and its human homologue. Nature 372: 425–432. 7984236 10.1038/372425a0

[3] Halaas JL, Gajiwala KS, Maffei M, Cohen SL, Chait BT et al (1995) Weight-reducing effects of the plasma protein encoded by the obese gene. Science 269: 543–546. 7624777 10.1126/science.7624777

[4] Myers MG, Cowley MA, Munzberg H (2008) Mechanisms of leptin action and leptin resistance. Annu Rev Physiol 70: 537–556. 17937601 10.1146/annurev.physiol.70.113006.100707

[5] Balland E, Cowley MA (2015) New insights in leptin resistance mechanisms in mice. Front Neuroendocrinol 39: 59–65. 10.1016/j.yfrne.2015.09.004 26410445

[6] Farooqi IS, O'Rahilly S (2005) Monogenic obesity in humans. Annu Rev Med 56: 443–458. 15660521 10.1146/annurev.med.56.062904.144924

[7] Lopez M, Tovar S, Vazquez MJ, Nogueiras R, Seoane LM et al (2007) Perinatal overfeeding in rats results in increased levels of plasma leptin but unchanged cerebrospinal leptin in adulthood. Int J Obes (Lond) 31: 371–377. 16801924 10.1038/sj.ijo.0803425

[8] Lopez M, Seoane LM, Tovar S, Garcia MC, Nogueiras R et al (2005) A possible role of neuropeptide Y, agouti-related protein and leptin receptor isoforms in hypothalamic programming by perinatal feeding in the rat. Diabetologia 48: 140–148. 15616803 10.1007/s00125-004-1596-z

[9] Contreras C, Gonzalez-Garcia I, Martinez-Sanchez N, Seoane-Collazo P, Jacas J et al (2014) Central ceramide-induced hypothalamic lipotoxicity and ER stress regulate energy balance. Cell Rep 9: 366–377. 10.1016/j.celrep.2014.08.057 25284795 PMC5157160

[10] Ottaway N, Mahbod P, Rivero B, Norman LA, Gertler A et al (2015) Diet-induced obese mice retain endogenous leptin action. Cell Metab 21: 877–882. 10.1016/j.cmet.2015.04.015 25980347 PMC4456263

[11] Guo DF, Cuim H, Zhang Q, Morgan DA, Thedens DR et al (2016) The BBSome controls energy homeostasis by mediating the transport of the leptin receptor to the plasma membrane. PLoS Genet 12: e1005890 10.1371/journal.pgen.1005890 26926121 PMC4771807

[12] Guo DF, Rahmouni K (2011) Molecular basis of the obesity associated with Bardet-Biedl syndrome. Trends Endocrinol Metab 22: 286–293. 10.1016/j.tem.2011.02.009 21514177 PMC3130119

[13] Novas R, Cardenas-Rodriguez M, Irigoin F, Badano JL (2015) Bardet-Biedl syndrome: Is it only cilia dysfunction? FEBS Lett 589: 3479–3491. 10.1016/j.febslet.2015.07.031 26231314

[14] Rahmouni K, Fath MA, Seo S, Thedens DR, Berry CJ et al (2008) Leptin resistance contributes to obesity and hypertension in mouse models of Bardet-Biedl syndrome. J Clin Invest 118: 1458–1467. 10.1172/JCI32357 18317593 PMC2262028

[15] Stratigopoulos G, Martin Carli JF, O'Day DR, Wang L, LeDuc CA et al (2014) Hypomorphism for RPGRIP1L, a ciliary gene vicinal to the FTO locus, causes increased adiposity in mice. Cell Metab 19: 767–779. 10.1016/j.cmet.2014.04.009 24807221 PMC4131684

[16] Starks RD, Beyer AM, Guo DF, Boland L, Zhang Q et al (2015) Regulation of Insulin Receptor Trafficking by Bardet Biedl Syndrome Proteins. PLoS Genet 11: e1005311 10.1371/journal.pgen.1005311 26103456 PMC4478011

[17] Thaler JP, Yi CX, Schur EA, Guyenet SJ, Hwang BH et al (2012) Obesity is associated with hypothalamic injury in rodents and humans. J Clin Invest 122: 153–162. 10.1172/JCI59660 22201683 PMC3248304

